# Is the measurement of tissue advanced glycosylation products by skin autofluorescence associated with mortality in patients treated by peritoneal dialysis?

**DOI:** 10.1007/s40620-022-01415-9

**Published:** 2022-08-18

**Authors:** Kornchanok Vareesangthip, Stanley Fan, Andrew Davenport

**Affiliations:** 1 Renal Division, Department of Medicine, Faculty of Medicine Siriraj Hospital, Mahidol University, Bangkok, Thailand; 2 Department of Renal Medicine, Royal London Hospital, Barts Health NHS Trust, London, UK; 3 UCL Department of Nephrology, Royal Free Hospital, University College London, Rowland Hill Street, London, NW3 2PF UK

**Keywords:** Peritoneal dialysis, Mortality, Autofluorescence, Diabetes, Malnutrition, AGEs

## Abstract

**Background:**

Advanced glycosylated end-products (AGEs) have been shown to cause cardiovascular disease, and tissue AGE accumulation can be measured by skin autofluorescence (SAF). AGEs are cleared by the kidney, and thus accumulate in dialysis patients. However, as the results of SAF measurements in peritoneal dialysis patients (PD) have been ambiguous, we examined the association between mortality and SAF.

**Methods:**

We reviewed SAF measurements in PD patients attending a university associated PD program, along with standard measurements of dialysis adequacy and peritoneal membrane function.

**Results:**

We studied 341 prevalent PD patients, 61.9% male, mean age 61.2 ± 16 years, and 31.4% of all patients died during a median follow-up of 27.2 (23.3–36.3) months. Patients who died were older, mean age 72 ± 10.5 years, were more often diabetic (60.7%), and had higher median SAF 3.8 (3.2–4.5) AU. On logistic regression, mortality was independently associated with age (odds ratio (OR) 1.1 (95% confidence limits 1.06–1.16), diabetes OR 10.1 (3.1–33.4), SAF OR 3.3 (1.8–6.2), all *p* < 0.001, and male gender OR 5.2 (1.6–17.4), *p* = 0.007; and negatively associated with weight OR 0.91 (0.86–0.95), *p* < 0..001, normalised nitrogen appearance rate (nPNA) OR 0.05 (0.01–0.4), *p* = 0.005 and mean arterial blood pressure (MAP) OR 0.96 (0.93–0.96), *p* = 0.03.

**Conclusions:**

In this observational study, SAF was independently associated with mortality. However, other factors were also associated with mortality, including age, diabetes and malnutrition which have all been reported to affect SAF measurements. Thus, the additional predictive value of measuring SAF compared to standard risk factors for mortality remains to be determined.

**Graphical abstract:**

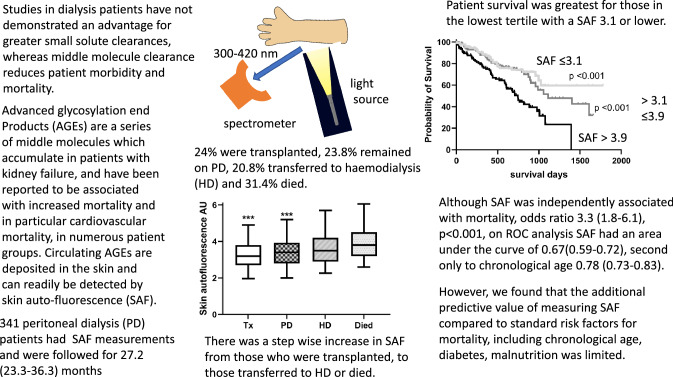

## Introduction

Patients with chronic kidney disease treated by dialysis have an increased risk of mortality, in particular cardiovascular mortality [1]. Increasing the amount of dialysis to achieve greater urea clearance has not been shown to improve patient survival [2–5]. However, more recent studies have suggested that clearance of larger sized, so-called middle molecules, improves patient survival [6], and reduces cardiovascular mortality [7]. This is supported by reports that patients with residual function, which increases the clearance of middle sized uraemic toxins, have improved survival [8, 9].

Advanced glycation end products (AGEs) are a series of middle-sized molecules which accumulate in patients with chronic kidney disease treated by dialysis. Serum AGEs, particularly the smaller sized AGEs, can be temporarily reduced following high-flux haemodialysis (HD) or haemodafiltration sessions, and as such, measurement of tissue AGEs by skin autofluorescence (SAF) provides a more reliable measurement [10]. AGEs are formed by the Maillard reaction and reflect cumulative metabolic oxidative stress, and induce chronic inflammation [11]. As such, tissue AGEs are thought to contribute to the progression of chronic, age-related diseases including atherosclerosis, and increased cardiovascular risk [12].

Studies in HD patients have suggested that mortality is increased in those patients with greater SAF [13, 14]. There have been very few reports in PD patients, as often observational studies have typically reported on a composite of HD and PD patients, with a majority of HD patients, and although one study suggested that patients with a higher SAF had increased mortality [15], another reported no such association [16]. In view of these different findings, we reviewed our own observational data to determine whether there is an association between SAF and patient outcomes.

## Patients and methods

We reviewed the data from PD patients attending two PD clinics, associated with a United Kingdom (UK) university, for routine outpatient review. Patients were treated with glucose containing dialysates, both standard single chamber glucose dialysates (Baxter Health Care, Deerfield, USA) and dual chamber dialysates (Fresenius Balance, Fresenius Medical Care, Bad Homberg, Germany, and Physioneal Baxter Health Care, Deerfield, USA).

SAF was also measured together with routine physical examination, body weight and mean arterial blood pressure (MAP) evaluation and laboratory investigations. SAF was measured in patients who had rested and were sitting in a room with a controlled temperature and no direct sunlight. The volar surface of the dominant arm was placed on the AGE reader (DiagnOptics, Groningen, Netherland).), avoiding discoloured and hairy areas. SAF was measured three times and the mean value was recorded in arbitrary units (Au). After each measurement the arm was repositioned to within 1 cm of the original placement. In brief, the AGE reader illuminates approximately 4 cm^2^ of the skin surface with an emission ultraviolet fluorescence light wave-length between 420 and 600 nm, which causes excitation of AGEs deposited in subcutaneous tissues, and then measures the autofluorescence-reflected light from the skin, wave length of 300–420 nm with a spectrometer [17, 18]. The AGE reader has a guard to prevent external light contamination [17, 18]. The AGE reader was fitted with an additional light source for those with darker skin pigmentation, and measurements were adjusted for skin colour by proprietary software (DiagnOptics, Groningen, Netherlands). All measurements were made by a single observer blinded to the patients’ clinical findings.

PD adequacy was calculated by standard methods from measurements of urea and creatinine in 24-h urinary collections and contemporaneous samples from 24-h spent PD dialysates [19]. We estimated normalised protein nitrogen appearance (nPNA) using standard equations [20]. Peritoneal membrane transport (PET) was calculated from a 4-h peritoneal dialysate dwell using a standard 2.0 L, 22.7 g/L peritoneal dialysate. We measured blood glucose, serum albumin using the bromocresol green method, while creatinine was measured enzymatically (Roche Modular P^®^ analyser, Roche Diagnostics Limited, Burgess Hill, UK) [21].

Patient demographics and laboratory investigations were retrieved from hospital computerised databases. We used the United Kingdom (UK) Stoke-Davies grading system for assessment of co-morbidity [22].

### Statistical analysis

Categorical data are presented as numbers (percentage) and continuous data are presented as mean ± standard deviation or median (interquartile range). Standard statistical tests were used to analyse data, i.e., D’Agostino & Pearson normality test, Student *t* test, Mann Whitney *U* test, Chi square (*X*^2^) analysis for categorical data, while Anova and Kruskal Wallis were used to compare numerical data between groups with appropriate corrections made for multiple testing (Tukey and Games Howell). Kaplan Meier and Cox regression were used to analyse both patient survival at the end of the study period and SAF, with patients divided into three groups according to SAF values. Variables associated with mortality were analysed using a step backward logistic regression model, with non-parametric variables log transformed, and variables then excluded if not statistically significant unless they improved the model fit. To review the relative effect of these variables on mortality, a series of receiver operator curves (ROC) were derived. Statistical analysis was carried out using Statistical Package for Social Science version 27.0 (IBM Corporation, Armonk, New York, USA), and Graph Pad (Graph Pad Prism version 9.2, San Diego, USA). Statistical significance was taken as *p* value < 0.05.

### Ethics

This observational study was registered with National Health Service (NHS) ethics and approved by a national research ethics committee (13/LO/0912), with informed consent to comply with the declaration of Helsinki. Retrospective analysis complied with the National Research Ethics Service audit procedures, with all data anonymised (audit registration number 12900).

## Results

**Fig. 1:**
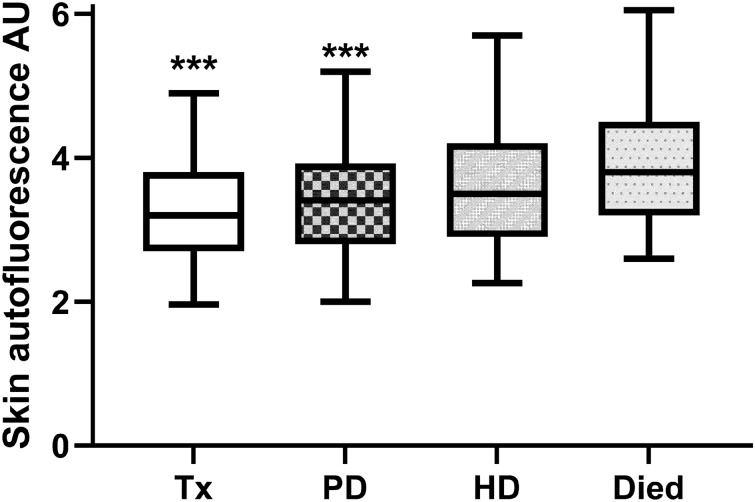
Skin autofluorescence in arbitrary units (AU) in those patients who were transplanted (Tx), remained on peritoneal dialysis (PD), transferred to haemodialysis (HD) or died. Median, interquartile range and 5% to 95% limits. ***p < 0.001 vs Died group

**Fig. 2:**
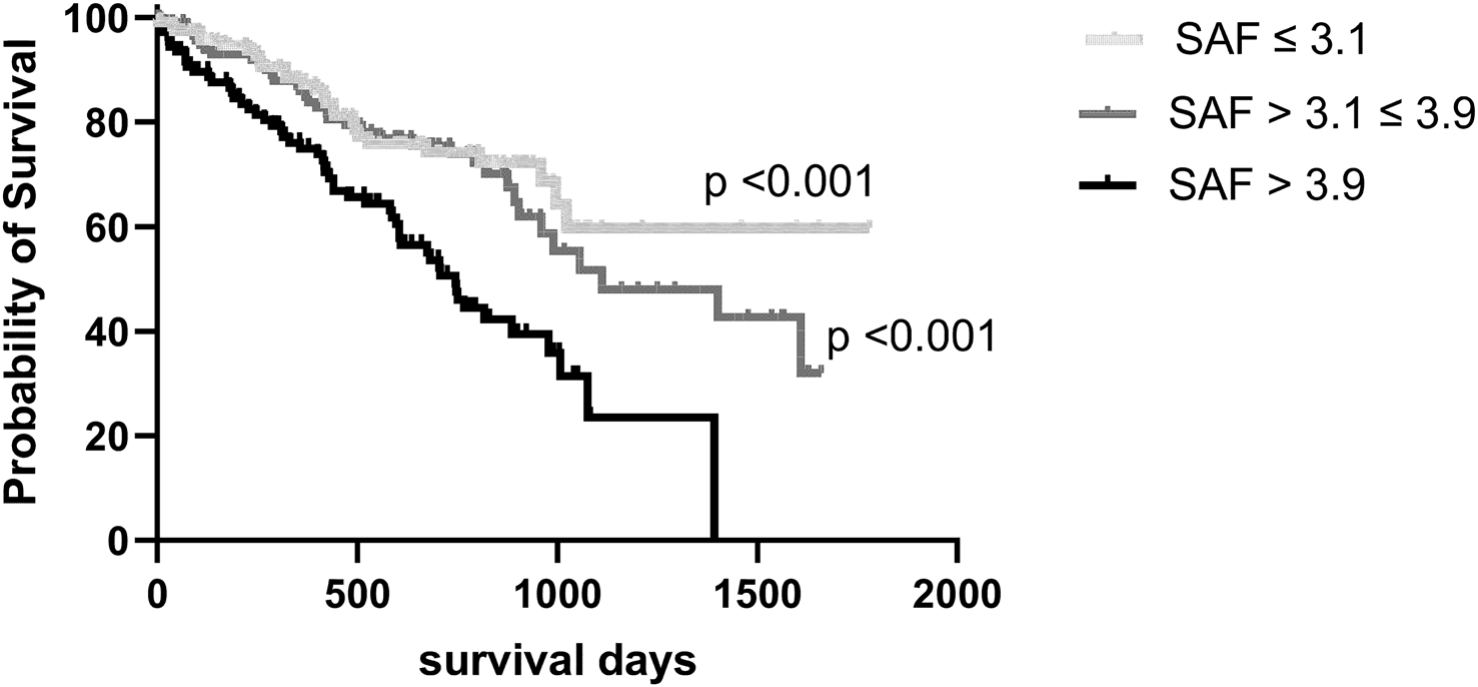
Patients divided into three groups according to Skin autofluorescence (SAF), lowest tertile ≤ 3.1 AU, middle tertile > 3.1 ≤ 3.9, and highest tertile > 3.9. Kaplan Meier Log rank analysis vs highest tertile

Three hundred and forty-one patients attending routine outpatient PD clinics between November 2016 and March 2020 were recruited and followed up until 5th May, 2022, median follow-up 27.2 (23.3–36.3) months. Patient demographics are set out in Table 1. The majority of patients were male and received overnight automated peritoneal dialysis with a day-time exchange (CCPD), and 45% were diabetic. On follow up, almost a third of patients had died (Table 2). Patients who died were older, had greater co-morbidity scores and were more likely to have diabetes (*X*^2^ = 17.7, *p* = 0.001), and greater co-morbidity (*X*^2^ 66.3, *p* < 0.001). However, there were no differences in PD treatment (use of continuous ambulatory peritoneal dialysis (CAPD), automated peritoneal dialysis with dry day (APD), CCPD, *X*^2^ 9.9, *p* > 0.05; or prescription of Baxter vs Fresenius dialysates, *X*^2^ 11.7, *p* > 0.05). SAF increased sequentially from those who were subsequently transplanted, remained on PD, transferred to haemodialysis or died (Fig. 1). Thus, patients who remained on PD had the longest treatment with PD, median 867 (796–1060) days, followed by those who died (418 (232–685) days), transferred to HD (407 (150–666) days) and those who were transplanted (391 (190–609) days). After dividing patients according to SAF, those in the tertile with the highest SAF had the greatest mortality when censored at the time of death or at the end of follow-up, as shown by log rank analysis (Fig. 2), and Cox proportional hazards (*z* = 3.73, *p* < 0.001).

**Table 1:** Patient demographics, peritoneal dialysis treatment, and investigations

Variable	
Number of patients	341
Male (%)	211 (61.9)
Diabetic (%)	153 (45)
Age years	61.2 ± 16.0
Months of peritoneal dialysis	10 (3.0–23.8)
CAPD/APD/CCPD %	31.1/19.1/49.9
Icodextrin exchange volume	1.1 (0–2)
22.7 g/L glucose exchange volume	0 (0–4.0)
Urine output mL/day	773 (382–1405)
Weekly Kt/Vurea	2.02 (1.6–2.53)
4 h D/Pcreatinine	0.72 (0.62–0.82)
nPNA g/kg/day	0.90 (0.96–1.11)
Weight kg	73.7 ± 15.7
Mean arterial blood pressure mmHg	101.1 ± 16.9
Serum albumin g/L	37.3 ± 4.7
Serum C reactive protein mg/L	4.0 (2.0–11.0)
Haemoglobin g/L	108.8 ± 15.1
Serum cholesterol mmol/L	4.47 ± 1.2
Blood glucose mmol/L	5.7 (4.9–9.1)
Serum urea mmol/L	20.4 ± 6.6
Serum creatinine umol/L	682 (514–873)
Davies co-morbidity score	1 (0–2)
Skin autofluorescence AU	3.63 ± 1.08

Continuous ambulatory peritoneal dialysis (CAPD), automated peritoneal dialysis (APD), automated peritoneal dialysis with day-time icodextrin exchange (CCPD), dialysis adequacy (Kt/Vurea), peritoneal membrane transport 4 h (D/Pcreatinine), normalised protein appearance rate (nPNA). Data expressed as integer, percentage, mean ± standard deviation, and median (inter-quartile range)

**Table 2:** Patients grouped according to follow-up. Transplanted (Tx), remained on Peritoneal dialysis (PD), transferred to haemodialysis (HD), and those who Died

Variables	Tx	PD	HD	Died
Number	82	81	71	107
Male (%)	49 (59.8)	44 (54.3)	50 (70.4)	68 (63.6)
Diabetic (%)	26 (31.7)	31 (38.3)	31 (43.7)	65 (60.7)
Age years	51.6 ± 14***	63.1 ± 15.3***	56.2 ± 16.9***	72.4 ± 10.5
PD vintage months	9 (3–19)	8 (2–14)	11 (4–26)	14 (3–25)
CAPD/APD/CCPD %	20/25.3/54.7	28.9/22.4/46.7	19.4/16.4/64.2	36.6/21.2/44.2
Icodextrin L/day	1.0(0–1.7)*	1.0(0–1.5)	1.5(0–2.0)	1.5(0–2.0)
22.7 g/L glucose L/day	0 (0–3.5)	0 (0–2.9)	1.3 (0–4.8)	2.0 (0–4.8)
Urine output mL/day	1000(644–1721)**	934(500–1741)*	618(185–1285)	600(200–1167)
Weekly Kt/Vurea	2.09(1.73–2.54)	2.11(1.65–2.84)	2.09(1.73–2.54)	1.95(1.58–2.46)
4 h D/Pcreat	0.68 ± 0.15	0.71 ± 0.14	0.74 ± 0.14	0.74 ± 0.12
nPNA g/kg/day	0.96 ± 0.24	0.99 ± 0.22**	0.94 ± 0.24	0.86 ± 0.25
Weight kg	76.1 ± 14.6	72.5 ± 15.8	77.7 ± 6.3***	70.0 ± 14.9
MAP mmHg	106.7 ± 12.0**	97.3 ± 20.8*	105.8 ± 15.0**	95.2 ± 16.2
Albumin g/L	38.9 ± 4.0***	38.7 ± 4.0***	37.0 ± 4.3*	35.2 ± 5.0
CRP mg/L	3.0(2.0–8.0)*	3.0(1.0–8.5)*	4.0(2.0–10.0)	7.0(2.0–19.0)
Haemoglobin g/L	106.2 ± 15.3	110.3 ± 14.3	106.8 ± 14.5	110.6 ± 15.6
Cholesterol mmol/L	4.6 ± 1.2	4.6 ± 1.3	4.5 ± 1.3	4.2 ± 1.1
Glucose mmol/L	5.3(4.6–5.6)***	5.6(5.0–8.8)	5.5(4.8–7.8)*	7.0(5.2–12.6)
Urea mmol/L	20.5 ± 5.8	20.6 ± 4.3	20.4 ± 5.8	19.8 ± 4.3
Creatinine umol/L	743(586–1008)***	616(492–828)	787(604–1088)***	579(472–773)
Co-morbidity score	0(0–1.0)***	1(0–1)***	1(0–1)***	2(1–2)

Months of PD treatment (vintage), Peritoneal dialysis adequacy (weekly Kt/Vurea), peritoneal membrane transport 4 h dialysate to plasma creatinine ratio (4hrD/Pcreat),), normalised protein appearance rate (nPNA), mean arterial blood pressure (MAP), C reactive protein (CRP),Stoke-Davies co-morbidity score (Comorbidity). Data expressed as mean ± standard deviation, and median (inter-quartile range). Comparison of numerical data *p < 0.05, ** < 0.01, *** < 0.001 vs Died group

Variables in the table were then entered into a step backward logistic regression model for mortality, checked for variable inflation factor and collinearity, with an adjusted model strength (*r*^2^) of 0.65. Mortality was independently associated with increasing age and SAF, male gender and diabetes, and negatively associated with increasing weight, nPNA, and MAP (Table 3). There was an association between age and SAF (*r*^2^ = 0.05, *p* < 0.001), and although diabetic patients had a higher SAF (3.7 ± 1.0 vs 3.4 ± 1.1 AU), *p* = 0.026, after adjustment for multiple testing this no longer remained statistically significant. Median SAF for both men and women were similar (3.5 vs 3.5 AU). To review the relative effect of these variables on mortality a series of ROC were derived. On ROC analysis, only age had a greater ROC area under the curve (AOC) than SAF with an area of 0.78 (95% confidence limits 0.73–0.83) compared to an area of 0.67 (95% confidence limits 0.59–0.72) with SAF. AUC for the other variables in the multivariable model were diabetes 0.62, weight 0.44, nPNA 0.41, and MAP 0.34, respectively. The paired sample difference between age and SAF was *z* = 1.99, *p* = 0.046, with a difference in the AOC of 0.099 (95% confidence limits 0.002–0.197). By adding a SAF of greater than 3.9, the upper SAF tertile, the ROC AUC for age increased to 0.81 (0.74–0.87).

**Table 3:** Logistic regression model of variables independently associated with mortality

Variable	*β*	StE β	Wald	Odds ratio	95% CI	*p* value
Age year	0.11	0.02	19.7	1.1	1.06–1.16	< 0.001
Diabetes	2.31	0.61	14.2	10.1	3.1–33.4	< 0.001
Weight kg	− 0.09	0.03	14.0	0.91	0.86–0.95	< 0.001
SAF AU	1.19	0.32	13.6	3.3	1.75–6.18	< 0.001
nPNA g/kg/day	− 3.07	1.1	7.8	0.05	0.01–0.4	0.005
Male	1.66	0.61	7.4	5.2	1.58–17.4	0.007
MAP mmHg	− 0.04	0.02	4.7	0.96	0.93–0.96	0.03

Standard error *β* (StE *β*), 95% confidence interval (95%CI), normalised protein appearance rate (nPNA), mean blood pressure (MAP). Adjusted model *r*^2^ 0.65

## Discussion

There have been a limited number of studies reporting on measurements of tissue AGEs by SAF in patients with chronic kidney disease treated by dialysis. The majority of these observational studies only examined HD patients [12–14], or if PD patients were included, they only made up a minority [16, 23]. Most of these studies reported that SAF readings were higher in HD patients who subsequently died, or in those with ischaemic heart disease [12]. One study in PD patients also reported an association between ischaemic heart disease and higher SAF readings [15], although another found that SAF measurements added little to the traditional risk factors for ischaemic heart disease [16].

We now report on SAF measurements from one of the largest PD cohorts to date and patient outcomes. There was a stepwise increase in SAF levels in patients who were subsequently transplanted, to those who remained on PD, transferred to HD and those who died. As centre policy was to provide a target amount of dialysis to achieve UK clinical guidelines in terms of the overall amount of urea clearance, overall clearances did not differ for patients who remained on PD, or transferred modality, or died [4]. However, SAF measurements were lowest in those who were subsequently transplanted, followed by those who remained on PD, then those who transferred to HD, and finally highest in those who died. These differences in SAF between the groups probably reflect that those who were transplanted were younger, healthier, and had fewer co-morbidities, while those who died were older and had more co-morbidities.

By dividing patients according to SAF, we observed that those in the upper tertile had significantly lower survival compared to the other groups. On logistic regression, increasing SAF was independently associated with mortality. Previous studies in both PD and HD patients have not established an association between survival and dialysis dose assessed by urea clearance [5, 8], whereas reports have suggested that greater clearance of middle molecules improved patient outcomes, and in particular that it reduced cardiovascular mortality [6, 7]. SAF reflects serum AGEs, a series of middle-sized molecules [14, 17] which cause vascular damage as they bind with receptors for advanced glycosylation end products (RAGEs), leading to increased oxidative stress and inflammation, thereby accelerating atherosclerotic damage and stiffening of arteries. Activation of RAGEs can induce complex signalling pathways leading to increased inflammation, oxidative stress and enhanced calcium deposition, and can alter the structure of low density lipoprotein, cross linking of collagen and elastin and increased vascular smooth muscle apoptosis, thus contributing to the development of both atherosclerosis and arteriosclerosis.

As such, our results would appear to support previous studies reporting an association between increased serum AGEs and SAF and both cardiovascular co-morbidity and mortality in HD and PD patients alike [12–15, 23–25].

As expected, mortality was associated with chronological age. SAF increases in the normal population with age [10], and, although statistically significant, we found that the association between chronological age and SAF was modest at 5%. This may have been due to the older age of our patient group, as studies including much younger patients (< 50 years) have shown that SAF increases with chronological age in dialysis patients [23]. However, when comparing our results with other studies, and after adjusting for age differences, our SAF readings are similar to those reported by several other groups [23]. Similarly, diabetes was associated with mortality, and although SAF is increased in diabetic subjects without kidney disease, results from observational studies in dialysis patients have varied as to whether SAF is greater in diabetic dialysis patients [12, 13, 23, 24, 26]. We found that SAF was higher in our diabetic patients, although not statistically significant after adjusting for multiple testing. In keeping with other studies in dialysis patients, mortality was also associated with lower body weight and reduced dietary protein intake as assessed by nPNA [27–29]. More recently, SAF has been reported to be increased in dialysis patients suffering from malnutrition [25]. Reports from outcome studies in PD patients have reported that male patients have greater mortality and greater PD technique failure compared to female patients [30]. In our cohort, male gender was also associated with mortality, although there were no differences in SAF between genders, whereas others have reported greater SAF measurements with men [23]. Patients with lower MAP were also found to be at increased risk of mortality, in keeping with other observational studies in PD patients [31]. This increased risk has been ascribed to underlying cardiovascular disease, and higher SAF values have been extensively linked to increased cardiovascular morbidity and mortality [12, 14–16, 23, 24].

Our observational report on almost 350 PD patients demonstrated an association between higher SAF and mortality. However, the time period between starting PD and SAF measurements varied between patients. Our results are in keeping with single centre studies reporting increased overall mortality, and cardiovascular mortality for HD patients and meta-analyses of studies in the general population, and in other at risk groups, including those with chronic kidney disease [32–34]. However, a review of other factors also associated with mortality, including chronological age, diabetes, malnutrition, gender, lower MAP and body weight, revealed that there is a potential association between SAF and these factors as well. On ROC analysis, SAF had an AUC of 0.67, which was lower than 0.78 observed for chronological age. Taking a higher SAF cut off value and combining it with chonological age only increased the AUC for the combination to 0.81. As such, our study raises the question as to the added value of measuring SAF in addition to recognised poor prognostic factors. This is in keeping with a study in pre-dialysis patients which reported that after adjustment for cardiovascular disease, diabetes, and other factors, SAF was no longer associated with mortality [34].

Although this is one of the largest studies reporting on outcomes of SAF measurements in PD patients to date, it is an observational study, and therefore can only report associations rather than attribute causality. Similarly, we only measured SAF on a single occasion and do not have longitudinal data. Nevertheless, while there was no association between weekly total urea clearance and outcomes, SAF was associated with mortality. However, the additional predictive value of measuring SAF compared to standard risk factors, including chronological age, diabetes and malnutrition has to be questioned.

## Conclusion

Advanced glycosylation end products, a series of middle molecules, can be measured by skin autofluorescence. Previous studies have reported an association between higher skin autofluorescence values and mortality in diabetic subjects, patients with cardiovascular disease and haemodialysis patients. In this observational study of almost 350 peritoneal dialysis patients, patients with a skin autofluorescence value above 3.9 had significantly reduced survival compared to those with lower values. Although skin autofluorescence was independently associated with mortality, other more conventional risk factors had stronger association with mortality.
